# Delineating the Seasonality of Varicella and Its Association With Climate in the Tropical Country of Colombia

**DOI:** 10.1093/infdis/jiad244

**Published:** 2023-06-29

**Authors:** Laura Andrea Barrero Guevara, Elizabeth Goult, Dayanne Rodriguez, Luis Jorge Hernandez, Benedikt Kaufer, Tobias Kurth, Matthieu Domenech de Cellès

**Affiliations:** Max Planck Institute for Infection Biology, Infectious Disease Epidemiology Group, Berlin, Germany; Institute of Public Health, Charité - Universitätsmedizin Berlin, Berlin, Germany; Max Planck Institute for Infection Biology, Infectious Disease Epidemiology Group, Berlin, Germany; Medicine Department, Universidad de los Andes, Bogotá, Colombia; Medicine Department, Universidad de los Andes, Bogotá, Colombia; Institute of Virology, Freie Universität Berlin, Berlin, Germany; Institute of Public Health, Charité - Universitätsmedizin Berlin, Berlin, Germany; Max Planck Institute for Infection Biology, Infectious Disease Epidemiology Group, Berlin, Germany

**Keywords:** chickenpox, childhood diseases, humidity, public health, transmission model

## Abstract

**Background:**

Varicella causes a major health burden in many low- to middle-income countries located in tropical regions. Because of the lack of surveillance data, however, the epidemiology of varicella in these regions remains uncharacterized. In this study, based on an extensive dataset of weekly varicella incidence in children ≤10 during 2011–2014 in 25 municipalities, we aimed to delineate the seasonality of varicella across the diverse tropical climates of Colombia.

**Methods:**

We used generalized additive models to estimate varicella seasonality, and we used clustering and matrix correlation methods to assess its correlation with climate. Furthermore, we developed a mathematical model to examine whether including the effect of climate on varicella transmission could reproduce the observed spatiotemporal patterns.

**Results:**

Varicella seasonality was markedly bimodal, with latitudinal changes in the peaks' timing and amplitude. This spatial gradient strongly correlated with specific humidity (Mantel statistic = 0.412, *P* = .001) but not temperature (Mantel statistic = 0.077, *P* = .225). The mathematical model reproduced the observed patterns not only in Colombia but also México, and it predicted a latitudinal gradient in Central America.

**Conclusions:**

These results demonstrate large variability in varicella seasonality across Colombia and suggest that spatiotemporal humidity fluctuations can explain the calendar of varicella epidemics in Colombia, México, and potentially in Central America.

## Teaser

The spatial variability in varicella seasonality across the tropical climates of Colombia may be explained by differences in the seasonal patterns of humidity across municipalities. This has implications for epidemic preparedness and vaccine program evaluations in tropical countries.

The varicella-zoster virus (VZV) causes varicella (chickenpox), a disease that afflicts over 70 million children every year worldwide [1]. Although the disease is typically mild and self-limiting, it can result in pneumonia, encephalitis, sepsis, and death (over 14 000 estimated deaths worldwide in 2019) [1], particularly in immunocompromised children [2]. After primary infection with VZV, the virus establishes latency in sensory neurons. Later in life, the virus can reactivate and cause shingles, a debilitating painful disease particularly prevalent in the elderly (2.9–19.5 cases per 1000 individuals over 50 years old) [3]. Hence, the large burden of varicella and shingles highlights the need to better characterize the epidemiology of varicella.

Similar to many other infectious diseases [4], the incidence of varicella is highly seasonal, with a latitudinal gradient in the peak timing observed on a global scale [5]. In temperate regions, this seasonality is typically characterized by a single peak at the end of winter (eg, in the United States and Germany) [6, 7], although 2 peaks have also been described (eg, in Japan and China) [8, 9]. The seasonal patterns in subtropical regions generally mirror those in temperate regions [10]. In tropical regions, however, varicella seasonality appears to be less definite, although observations have remained scarce because of a lack of epidemiological surveillance [11]. Bridging this data gap in tropical regions is crucial for 2 reasons. First, most of the morbidity and mortality associated with varicella affect low- to middle-income countries, many of which have a tropical climate [1]. Second, the climate in tropical regions—with almost constant temperature but variable precipitation and humidity during dry and rainy seasons—offers an opportunity to evaluate how different climatic variables impact varicella transmission, expanding on previous studies in temperate regions [8, 12]. A better understanding of varicella seasonality in a given setting may support public health efforts, informing epidemic preparedness and infection control.

In this study, we aimed to characterize the calendar of varicella epidemics across the climatically diverse regions of Colombia, where exhaustive surveillance has been in place for several years. Using a combination of statistical and mathematical models, we find that spatiotemporal variations of humidity may explain the calendar of varicella epidemic across Colombia and México, and we predict the existence of a spatial gradient of varicella seasonality in Central American countries. This finding may have implications for epidemic preparedness and evaluating the impact of vaccination programs.

## METHODS

### Data Sources

#### Varicella Data

We gathered weekly varicella data for each municipality in Colombia using publicly available, clinically confirmed notifications from the Colombian national surveillance system (SIVIGILA) (Supplementary Data). We focused on the 2011–2014 period; we excluded earlier data to minimize reporting bias caused by the gradual introduction of the SIVIGILA after 2007 and later data because of the varicella vaccine introduction in 2015 [13]. In addition, we focused on children ≤10 years because most cases of varicella typically occur in this age group. To avoid age misreporting, we excluded cases in which the reported age differed from that calculated from the birthdate.

Colombia is divided into 1122 municipalities, administrate divisions that are roughly equivalent to counties. Because many municipalities had low case counts, for definiteness, we selected the municipalities with a signal-to-noise ratio (mean to standard deviation [SD] ratio of the weekly reports) above 1 and an average of at least 5 varicella cases per week (Supplementary Figure 1).

#### Climate Data

Weekly mean temperature (unit, °C), specific humidity (g/kg), and relative humidity (%) data were extracted from the 32-km grid North American Regional Reanalysis (NARR) [14] dataset, and the total weekly precipitation was extracted from the 10-km grid Climate Hazards Group InfraRed Precipitation with Station (CHIRPS) [15] dataset for each municipality. In addition, we calculated the absolute humidity (g/m^3^) from the relative humidity (Supplementary Figure 2) considering the altitude of the municipalities [16]. For México and Central America countries (Panamá, Costa Rica, Nicaragua, Honduras, El Salvador, Guatemala, and Belize), we obtained the specific humidity from the NARR for the capital city of every first-level subnational division (Supplementary Data).

### Descriptive Model of Varicella Seasonality

To describe the spatial variability in varicella seasonality across Colombia, we fitted generalized additive models (GAMs) to the weekly incidence data. These flexible extensions of generalized linear models allow the modeling of complex, potentially nonlinear associations using smooth functions of predictor variables while preventing overfitting by penalizing the wiggliness of the function [17]. The base model included 2 predictors: a cyclic spline term to model the seasonality in each municipality and a municipality-year parametric intercept to capture the yearly average incidence in each municipality. We used a negative binomial model (with a log link) that included the log-transformed population size as an offset, so that the modeled outcome was the incidence rate. To model the spatial variability in varicella seasonality, we included a tensor product smooth between the week number and latitude with 2 smoothing bases: a cyclic spline for the week number and a cubic regression spline for the latitude. For all models, we used maximum likelihood to estimate the parameters of all models [18], and we calculated the Akaike information criterion (AIC) to compare their parsimony.

### Correlation Between Varicella Seasonality and Climatic Variables

We aimed to assess the correlation between the spatial variability of varicella seasonality and that of climates across Colombia. However, we did not attempt to directly regress against meteorological variables because seasonal forcing in transmission and long-term changes in population immunity can result in complex, nonlinear incidence dynamics that may not be captured by standard regression models [19]. Instead, we first calculated the dissimilarity matrix (using the Euclidean distance between standardized time series) of varicella data and of each climate variable between municipalities across Colombia. We also calculated the between-municipality dissimilarity matrix of the following sociodemographic factors: population density, population density of children ≤10 years old, migration, and urbanization.

We used the dissimilarity matrices to cluster the municipalities and evaluate the correlation between the climatic variables and varicella. We applied the partitioning around medoids algorithm for clustering, and we estimated correlation between dissimilarity matrices using Mantel tests.

### Transmission Models of Varicella Seasonality

To further test and formalize the hypothesis that climatic variables explain the seasonality of varicella in Colombia, we developed a simple compartmental transmission (SEIR) model (Supplementary Methods), in which the transmission rate of varicella infection was seasonally forced by changes in contact rates due to alternating school terms and school vacations (ie, term-time forcing) and by climate. Specifically, term-time forcing was modeled by a square wave whose amplitude was fixed based on the empirical observation that schoolchildren make 40% fewer contacts during school holidays than during school terms [20]. In sensitivity analyses, we also tested 2 additional scenarios: 1 with lower amplitude and 1 without term-time forcing. For climate forcing, we assumed an exponential relationship between the transmission rate and the standardized climatic variable. This relationship was scaled by an amplitude parameter, whose value was approximately calibrated to reproduce the observed seasonality of varicella across Colombia. The model was represented as a set of differential equations describing the movement of individuals between the compartments. Furthermore, we used the same model together with data from climatic variables from México and countries in Central America to predict varicella seasonality in these countries.

## RESULTS

### Incidence of Varicella in Colombia Between 2011 and 2014

**Figure 1.**
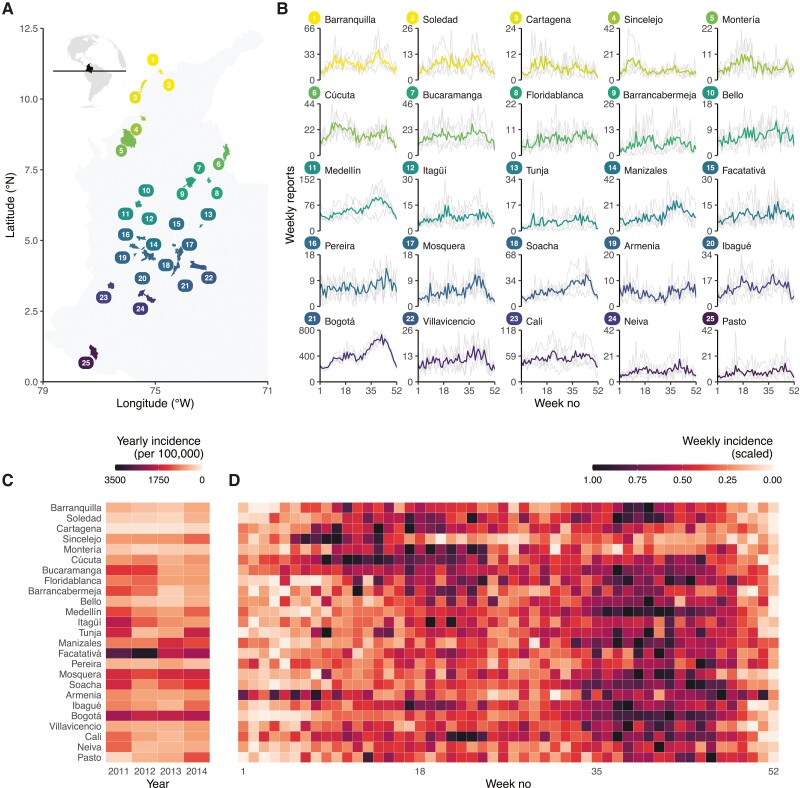
Varicella reports and incidence of varicella in Colombia, 2011–2014. (A) Map of included municipalities. (B) Mean weekly varicella reports in the municipalities of Colombia: lighter lines show the weekly reports every year. (C) Mean yearly varicella incidence per 100 000 children ≤10 years of age per municipality, and (D) mean weekly varicella incidence of varicella per municipality (rescaled so that 0 is the minimum and 1 is the maximum number of cases per municipality). From bottom to top, the municipalities are ordered by increasing latitude.

Between 2011 and 2014, a total of 421 085 varicella cases were reported in Colombia. Once cases with missing or misreporting age were excluded (0.018% and 0.097%, respectively) (Supplementary Figure 3), children ≤10 years accounted for 55.5% of the total cases throughout the country. After applying the municipality selection criteria, the final dataset comprised 156 976 cases in 25 municipalities (67.4% of all the cases in children up to age 10), which spanned a large area and range of climates of Colombia (latitude, 1.2–11.0° N; longitude, 72.5–77.3° W; and 6 of the 17 Köppen-Geiger [21, 22] climate classifications in the tropics (Figure 1*A* and Supplementary Figure 1).

Most varicella cases were reported in Bogotá (56.1% of the total), followed by Cali (8.30%) and Medellín (7.06%), the 3 most populated municipalities in Colombia (Figure 1*B*). The yearly reported incidence varied substantially (range, 3291.8 cases per 100 000 children in Facatativá in 2012 to 113.9 cases per 100 000 children in Cartagena in 2012). The high incidence in Facatativá may be explained by its proximity to Bogotá (Figure 1*C*). However, the mean age of infection was similar across municipalities, ranging from 4.6 to 5.5 years (Supplementary Figure 3).

### Latitudinal Gradient Demonstrates Substantial Spatial Heterogeneity of Varicella Seasonality Across Colombia

**Figure 2.**
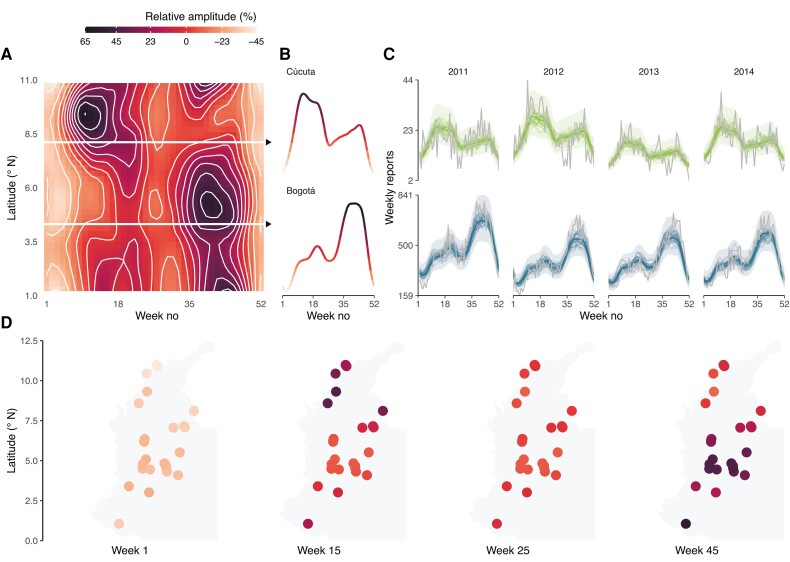
Latitudinal gradient of varicella seasonality across Colombia estimated from generalized additive models (GAMs). (A) The shape of the partial effects from the seasonality (relative amplitude [%] from the mean) for varicella incidence across latitudes. (B) Estimated seasonality in Cúcuta (7.9° N latitude) and in Bogotá (4.7° N latitude). (C) Observed (lighter lines) and predicted (darker lines and envelopes) seasonality of varicella reports in Bogotá and Cúcuta throughout the study period. The lines represent 30 random draws from the model posterior, and the ribbons represent an approximate 95% simultaneous confidence interval for the fitted GAM. (D) Spatial variability in the varicella seasonality (relative amplitude) across municipalities at the beginning (week 1) and middle of the year (week 25) and at the varicella peaks (weeks 14 and 45).

The incidence of varicella was markedly seasonal across Colombia, with an early peak at approximately week 15 more pronounced in the northern municipalities and a late peak at approximately week 40 more pronounced in the southern municipalities. Hence, the amplitude of both peaks changed substantially with latitude (Figure 1*D*). The best-fitting GAM confirmed the spatial variability in varicella seasonality, with strong statistical evidence of a latitudinal gradient (ΔAIC = 309 compared with a model with no spatial variability) (Figure 2*A* and Supplementary Figure 4). The early peak was more pronounced than the late peak in northern (latitude, 7.0–11.0° N) municipalities, for example, in Cúcuta (7.9° N latitude) (Figure 2*B* and *C*). By contrast, the late peak had higher amplitude in southern (latitude: 1.2–7.0° N) municipalities, such as Bogotá (4.7° N latitude) (Figure 2*B* and *C*). The best-fitting model performed well at reproducing the observed data in every municipality (R-squared range, 71.9% in Soacha to 98.5% in Mosquera) (Figure 2*D*), with little evidence of residual autocorrelation (Durbin-Watson statistic = 0.35, *P* = .49). Hence, our descriptive model accurately recapitulated the spatiotemporal dynamics of varicella and evidenced substantial spatial heterogeneity of varicella seasonality across Colombia.

### Spatial Variability of the Varicella Seasonality Correlates With Variability in Climates Across Colombia

**Figure 3.**
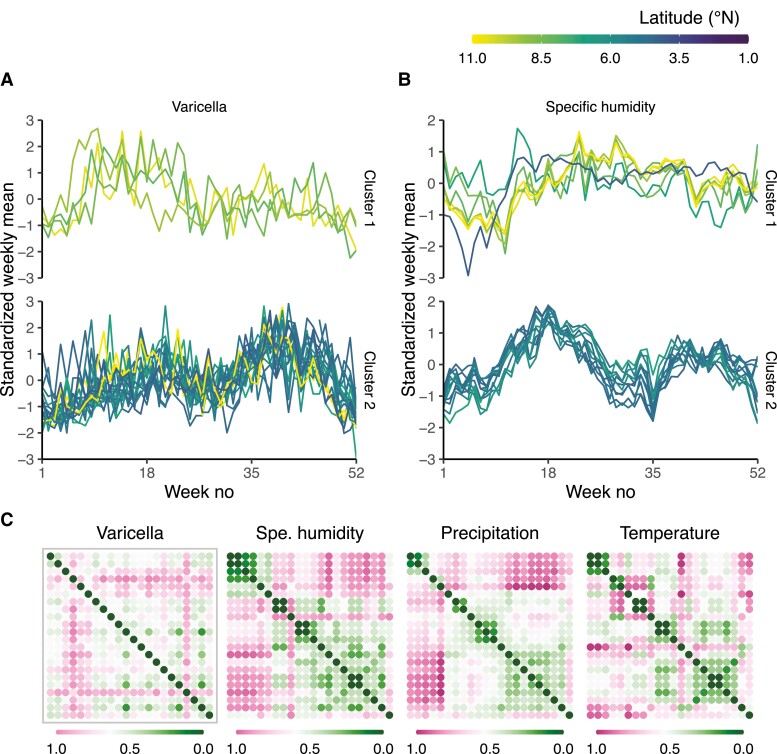
Correlation between spatial heterogeneity of varicella seasonality and that of climatic variables. Standardized weekly mean varicella reports (A) and specific humidity (B) independently clustered in 2 municipality groups. (C) Between-municipality dissimilarity (based on Euclidean distance) matrices of varicella, specific humidity, precipitation, and temperature (from bottom to top and left to right, the municipalities are ordered by increasing latitude).

The latitudinal gradient described above suggests that spatial heterogeneity of climates could explain the observed seasonality of varicella across Colombia. We formalized this hypothesis of a causal framework using a directed acyclic graph (DAG), in which latitude explained the between-municipality distance and variability in climate, which itself explained the variability in varicella seasonality (Distance←Latitude→Climate→Varicella) (Supplementary Figure 5). To test this hypothesis, we first applied clustering methods to identify groups of municipalities with broadly similar varicella seasonality. In keeping with our previous observations, we found evidence for 2 clusters (Hopkins statistic = 0.69): one including northern municipalities with a more pronounced early peak and the second including southern municipalities with a more pronounced late peak (Figure 3*A*). Next, we clustered the data for each climate variable (Figure 3*B*). For specific humidity, absolute humidity, and precipitation, we found evidence for 2 clusters (Hopkins statistic = 0.75, 0.74 and 0.79) (Supplementary Figure 6), which broadly matched the 2 clusters of varicella seasonality (fraction of matching pairs: 81.8%, 81.8%, and 95.5%). By contrast, the temperature and relative humidity variables clustered into more than 2 groups that did not match those of varicella seasonality (Supplementary Figure 6).

To strengthen the evidence for a link between climate and varicella seasonality, we ran a series of Mantel tests to assess the correlation between the spatial dissimilarity matrix of varicella seasonality and that of every climate variable (Figure 3*C*). We found evidence of strong positive correlations with all humidity variables and precipitation but not with temperature (Table 1). We also ran additional analyses to test our assumption that distance between municipalities does not directly affect varicella seasonality (Supplementary Figure 5). Despite evidence of a positive correlation with distance from a direct Mantel test (Mantel statistic = 0.278, *P* = .017), this correlation became negligible after correcting for latitude or humidity in a partial Mantel test (Mantel statistic = 0.132 and −0.002, *P* = .131 and *P* = .456, respectively). Moreover, the correlation with humidity remained robust after controlling for latitude or distance (Mantel statistic = 0.316 and 0.337, *P* = .003 and *P* = .003, respectively). In addition, we found no evidence of a latitudinal gradient in the considered sociodemographic factors (Supplementary Figure 7) and, therefore, no correlation with the latitudinal gradient in varicella seasonality (Supplementary Table 1). These results suggest that spatial variability in climate across municipalities, but not the spatial distance between municipalities, can explain the variation in the seasonality of varicella across Colombia.

**Table 1.** Correlation Between the Spatial Heterogeneity of Varicella Seasonality and that of Climate^a^

Variable Tested	Control Variable	Mantel Statistic	*P* Value
Specific humidity	…	0.412	.001
Absolute humidity	…	0.398	.001
Total precipitation	…	0.319	.008
Relative humidity	…	0.244	.020
Mean temperature	…	0.077	.225
Specific humidity	Distance	0.316	.003
Specific humidity	Latitude	0.337	.003
Distance	…	0.278	.017
Distance	Latitude	0.132	.131
Distance	Specific humidity	−0.002	.456

The correlations were calculated using Mantel and partial Mantel tests on the dissimilarity matrices which were calculated using Euclidean distances between the time series of paired municipalities.

### A Mechanistic Model With Transmission Rate Seasonally Forced by Humidity Can Reproduce the Latitudinal Gradient of Varicella Seasonality in Colombia

**Figure 4.**
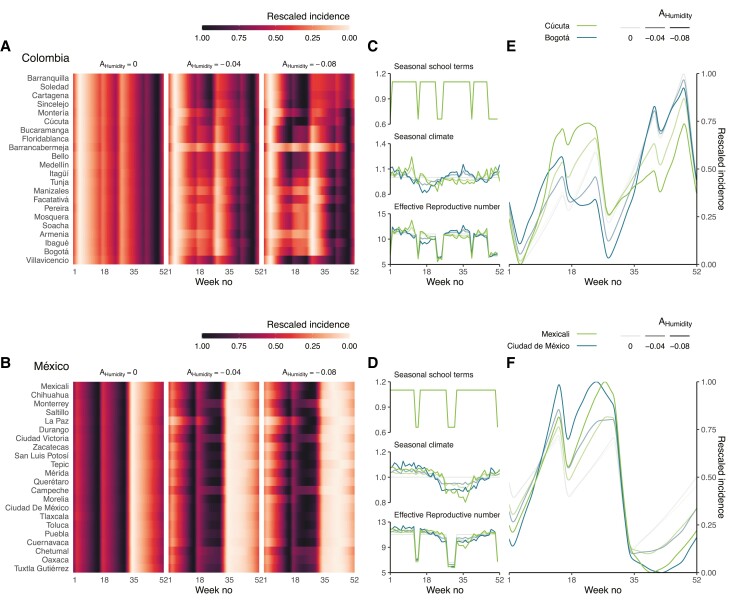
Transmission models predicting the impact of seasonal humidity and alternating school terms and holidays on the transmission rate can reproduce the latitudinal gradient of varicella seasonality in Colombia and México. Predicted varicella incidence (rescaled so that 0 is the minimum and 1 is the maximum number of cases per municipality) for all (A) municipalities in Colombia and (B) cities in México. (C and D) Estimated seasonal components of the transmission rate and (E and F) corresponding seasonal varicella profiles in the municipalities of Cúcuta (7.9° N latitude) and Bogotá (4.7° N latitude) and in the cities of Mexicali (32.6° N latitude) and Ciudad de México (19.4° N latitude). From bottom to top, the municipalities are ordered by increasing latitude. For all municipalities and cities, the model was run for a period of 200 years until equilibrium. The results displayed correspond to the last simulated year.

To further examine the impact of climate on varicella seasonality in Colombia, we formulated a simple mechanistic model in which the transmission rate of varicella infection was seasonally shaped by alternating school terms and school holidays (ie, term-time forcing) [23] and by variations of specific humidity (ie, humidity forcing). In the absence of humidity forcing (Figure 4*A*, left heatmap), the incidence of varicella gradually increased after the Christmas and mid-year vacations, resulting in a first peak at approximately week 24 and a second, more pronounced peak at approximately week 49 throughout Colombia. As expected, however, this scenario failed to recreate any latitudinal gradient. By contrast, a latitudinal gradient was predicted by simulations with a small amplitude of humidity forcing (where a 1-SD increase in humidity resulted in a 4% decrease in the transmission rate of varicella infection) (Figure 4*A*, middle heatmap). Increasing the effect of humidity (with amplitude fixed to −8%) forcing resulted in a more definite latitudinal gradient, which broadly reproduced that observed in Colombia, with a contrast between southern and northern municipalities (Figure 4*A*, right panel, and C and E).

When decreasing the amplitude of the school term-time forcing, the model underestimated the amplitude of the second peak in all municipalities and overestimated that of the first peak in the southern municipalities (Supplementary Figure 8). Hence, both term-time forcing and humidity forcing were required to reproduce the spatiotemporal variations in varicella across Colombia.

To complement our Colombian data, we simulated the dynamics of varicella in México, a subtropical country with variable climates but uniform subnational seasonal variations of humidity across 32 cities. In stark contrast to Colombia, the predicted seasonality of varicella did not vary across space, with a single peak at approximately week 14 in all cities, as observed previously (Figure 4*B*, *D*, and *F* and Supplementary Figure 9) [24]. To further predict the importance of subnational heterogeneity of climates, we simulated varicella dynamics in all Central American countries. In the northern countries of Belize, Guatemala, and El Salvador (located south of México), we predicted only small variations of varicella seasonality between the different cities. In the southern countries of Panamá, Costa Rica, Nicaragua, and Honduras, however, we predicted substantial subnational variability in varicella seasonality, echoing our findings in Colombia (Supplementary Figure 10). These results emphasize the need to pay careful attention to the subnational heterogeneity of climates when studying the seasonality of varicella.

## DISCUSSION

The main purpose of this study was to describe the seasonality of varicella and its association with climate across the tropical country of Colombia. We found evidence for a latitudinal gradient in the peak magnitude of the seasonality of varicella that correlated with spatiotemporal variations of humidity. This heterogeneity can be explained by the wide diversity of climates in Colombia, attributable to a unique combination of the Intertropical Convergence Zone, the Andes Mountains, and the South American monsoon in this country [25]. In contrast, we predicted that the spatially uniform humidity profiles of México resulted in rigidly unimodal varicella seasonality throughout the country's subtropical climates. These predictions are confirmed by previous observations in México [24, 26]. Together with our predictions across Central America, these results suggest that varicella seasonality is less spatially homogeneous in tropical countries of this region. A practical consequence is that the observed epidemiological dynamics of varicella may be blurred in nationwide reports, which have been used in previous studies to characterize varicella seasonality worldwide and to estimate the impact of the introduction of the varicella vaccine (2015) in Colombia [5, 27]. These results echo earlier recommendations made for other pathogens, such as rotavirus and severe acute respiratory syndrome coronavirus 2 [28–30]. Therefore, our results show the need to carefully consider the subnational heterogeneity of varicella for epidemiological studies and public health policy.

Beyond tropical regions, the seasonality of varicella varies with latitude globally, with a peak in temperate regions during March–May in the northern hemisphere and during October–December in the southern hemisphere [5]. Although a single peak is observed in most temperate countries (like in many European countries, the United States, Australia, and South Africa), 2 peaks are observed in other countries at similar latitudes (like the United Kingdom, Japan, and China) [8, 31, 32]. Previous modeling studies have attributed these patterns to seasonal variations in host contacts [33–35], temperature [8, 12], and less frequently to precipitation [9] and humidity [24]. In temperate regions, the impact of humidity can be difficult to assess because it is highly correlated with temperature [36]. In contrast, tropical climates offer a quasi-experimental setting where humidity, but not temperature, varies seasonally (Supplementary Figure 2). Hence, our findings add to the body of evidence on the impact of humidity on varicella transmission dynamics. Emphatically, our finding that spatiotemporal variations of humidity best explained the variability in varicella seasonality across municipalities does not rule out the contribution of other seasonal drivers to the temporal variability of varicella within municipalities. Indeed, even though the school calendar varied little across municipalities, we found a clear signature of term-time forcing in our dataset (Supplementary Figure 8). Hence, this finding aligns with earlier evidence of the central role of term-time forcing in the seasonal epidemiology of varicella [34, 35]. Similarly, our results do not rule out the effect of other climatic variables (like temperature), but they suggest that their individual contribution may vary with latitude. More generally, we propose that together with other elements affecting transmission, like vaccination, seasonal variations in host contacts and climate can jointly explain the global seasonality of varicella.

Our findings suggest that humidity can impact the transmission of varicella and shape part of its seasonality. Climatic variables may impact pathogen transmission through multiple biological mechanisms that can affect the pathogen, the individual host, or the host population [19, 30, 37]. At the pathogen level, experimental evidence has shown that humidity and temperature can impact the formation and diffusion of aerosols and viral stability [4]. Dry weather would facilitate the formation and dispersion of aerosols, whereas cold weather would prolong the stability period of the virus [38, 39]. Nevertheless, the humidity and temperature range optimal for aerosol formation and viral stability vary markedly between different viral species [4], highlighting the need for experimental studies on VZV. At the individual host level, changes in humidity may alter the host physiology, for example, the integrity of the respiratory mucosa, as demonstrated for influenza A in mice [40]. Finally, at the host population level, variations of humidity and precipitation—in particular during rainy seasons—might modify the frequency of social contacts [41, 42]. Hence, further studies will be needed to test the biological mechanisms described above and to elucidate how humidity might affect varicella transmission.

Several limitations of our study are worth noting. First, varicella reporting in the surveillance system of Colombia might lack specificity because it includes zoster cases in addition to varicella cases. Nevertheless, we reduced this possible misclassification by focusing on children ≤10 years, in whom zoster is extremely rare [3]. Second, because of its observational design, further experimental studies are warranted to unveil potential causal effects. Even though we used a DAG to formalize our causal assumptions, we may have overlooked other explanations for the seasonality of varicella. Hence, further experimental studies in animal models and epidemiological studies applying causal inference methods for time series [43] will be useful to confirm our findings. Third, in our transmission model, we assumed an exponential relationship between humidity and the transmission rate of varicella. This relationship has often been used because it is easily interpretable [44], but other relationships, such as power functions, are possible [24]. Future work could estimate the form of this relationship, for example, by using recently developed statistical inference methods to confront transmission models to incidence data [45]. Fourth, in contrast with other northern municipalities, the seasonality of varicella in Barranquilla and Soledad displayed 2 peaks with similar amplitudes. Further studies could aim to understand regional characteristics that explain this pattern, which was not well captured by our model. Finally, because of our focus on Latin America, our results may not generalize to tropical regions in other parts of the world. In particular, it would be especially informative to expand our study to tropical regions of Africa or Asia, where varicella underlying transmission dynamics may be substantially different [1].

## CONCLUSIONS

In conclusion, our results demonstrated substantial variability in varicella seasonality across the tropical climates of Colombia. They further suggested that seasonal variations of humidity and host contact rates could capture most of the spatiotemporal dynamics of varicella incidence in this country. The predictable occurrence of varicella during specific times of the year is important for epidemic preparedness. In addition, careful consideration of seasonal heterogeneity may be crucial for unbiased estimation of the impact of varicella vaccination programs, which were recently rolled out in Colombia. More generally, these results may be helpful for future research on the epidemiology of varicella in Colombia and other tropical countries.

### Data Sharing

All of the data are freely available on the web pages of the SIVIGILA, IDEAM, NARR, and CHIRPS. Furthermore, all of the aggregated data (to the level of municipality-week) and code are stored in Edmond, the open research data repository of the Max Planck Society, to ensure the reproducibility of the results and are available online; no end date (https://doi.org/10.17617/3.ZCMEKJ). R version 4.1.2 (2021-11-01) was used for all analyses. The climate data were obtained using the packages “ncdf4”, “chirsp”, “humidity”, and “kgc” [16, 22, 46, 47]. Colombia map figures were created using the package “colmaps” [48]. The package “mgcv” was used for all the GAMs estimations [17]. The dissimilarity matrices, clusters, and mantel tests were obtained with the packages “TSclust” and “vegan” [49, 50]. The simulations were performed using the “pomp” package [45].

## Supplementary Data

Supplementary materials are available at *The Journal of Infectious Diseases* online. Consisting of data provided by the authors to benefit the reader, the posted materials are not copyedited and are the sole responsibility of the authors, so questions or comments should be addressed to the corresponding author.

## Supplementary Material

jiad244_Supplementary_Data

## Notes

***Financial support***. This work was supported by the Max Planck Society (Berlin, Germany). Funding to pay the Open Access publication charges for this article was provided by the Max Planck Society.
