# Supplementary material for: Delineating the Seasonality of Varicella and Its Association With Climate in the Tropical Country of Colombia
Source: J Infect Dis. 2023 Jun 29;228(6):674–83. doi: 10.1093/infdis/jiad244 (PMC10503957; doi:10.1093/infdis/jiad244)
Supplement: jiad244_Supplementary_Data [file jiad244_supplementary_data.pdf]

Supplementary Material - Delineating the seasonality of varicella and its association with climate in the tropical country of Colombia

Contents

S1 Supplementary data S2
S2 Supplementary methods S2
S3 Supplementary figures S4
References S12

Laura Andrea Barrero Guevara[1,2,*], Elizabeth Goult[1], Dayanne Rodriguez[3], Luis Jorge Hernandez[3], Benedikt Kaufer[4], Tobias Kurth[2], Matthieu Domenech de Cellès[1]

1. Max Planck Institute for Infection Biology, Infectious Disease Epidemiology group, Charitéplatz 1, Campus Charité Mitte, 10117 Berlin, Germany
2. Institute of Public Health, Charité – Universitätsmedizin Berlin, Charitéplatz 1, 10117 Berlin, Germany.
3. Medicine Department, Universidad de los Andes, Bogotá 111711, Colombia
4. Institute of Virology, Freie Universität Berlin, Robert-von-Ostertag-Str. 7, 14163 Berlin, Germany

*Corresponding author: Laura Andrea Barrero Guevara, Max Planck Institute for Infection Biology, Charitéplatz 1, Campus Charité Mitte, 10117 Berlin, Germany. E-mail address: barrero-guevara@mpiib-berlin.mpg.de

---

S1 Supplementary data

S1.1. Varicella data

In Colombia, healthcare institutions report clinically confirmed varicella cases to the national surveillance system (*Sistema Nacional de Vigilancia en Salud Pública*, SIVIGILA), which in turn publishes online the freely available unidentifiable individual data [1,2]. The SIVIGILA works under the Colombian National Health Institute (NHI) in cooperation with the Pan American Health Organization (PAHO) and collects weekly data from public and private healthcare provider institutions. According to "Law 1712 of 2014 for Transparency and the Right to Access Public Information", the SIVIGILA database is available publicly online. The database provides 1) aggregated data, consisting of municipality-level, weekly time series of varicella cases, and 2) individual data without personal identification information from 2007 to date [3,4]. We aggregated the individual data by week and municipality, and all the analyses were performed using the aggregated data, which is available in the Edmond repository.

A varicella case is clinically defined by the SIVIGILA as an acute onset illness that initiates with moderate fever and small erythematous macules that evolve into papules, water-clear vesicles, yellowish pustules, and finally, crusts. The cases are evaluated by a healthcare professional and can be epidemiologically linked to another case. A varicella case is then stored under the 831 code in the SIVIGILA database.

S1.2. Demographic data

We obtained municipality-level data on the population using demographic estimates from the Colombian National Administrative Department of Statistics (DANE) [5]. With these estimates and the area of each municipality, we estimated the population density and population density of children ≤10 years old from 2011–2014 per municipality. To assess migration and urbanization in each municipality, we examined the fraction of the total varicella cases reported in children who are migrants and the fraction of varicella cases reported in children residing in urban areas.

S1.3. Spatial resolution and coordinates

The spatial resolution was defined at the level of the municipalities for Colombia, considering that the varicella data is reported at the same level. For México and Central America, the analyses were performed for the capital city of the first-level administrative divisions of each country. For Colombia, coordinates of the centroid for each municipality were obtained from the Colombian National Institute for Hydrology, Meteorology, and Environmental Studies (IDEAM) database [6]. For México and Central America, coordinates of the capital cities were obtained from Google Maps.

S1.4. School terms and school holidays data

We obtained the school information from 2014 for Colombia from the Education Ministry website [7]. Data for Mexico and other Central American countries (Panamá, Costa Rica, Nicaragua, Honduras, El Salvador, Guatemala, and Belize), were obtained likewise [8–14].

S2 Supplementary methods

S2.1. Transmission model formulation

We formulated a Susceptible-Exposed-Infected-Recovered (SEIR) model of varicella transmission, including school terms and climate as sources of seasonality for the transmission rate. The term-time forcing driven by the alternation between school terms and school holidays was modeled using a square wave:

$$S_1(t) = \frac{1 + A_1 Term(t)}{1 - A_1(1 - 2p_{school})}$$

Where Term(t) equals 1 during school terms and –1 during school holidays. The parameter A_1 represents the amplitude of term-time forcing, fixed to 0.25 based on the empirical observation that schoolchildren make 40% fewer contacts during holidays than during school terms ($(1 - A_1)/(1 + A_1) = 0.6$) [15]. The denominator represents a correction factor, used to ensure that the square wave has a seasonal mean of 1. The climate forcing was modeled as:

$$S_2(t) = e^{A_2 Climate(t)}$$

where Climate(t) is the weekly standardized specific humidity, and A_2 represents the amplitude of the climate forcing, fixed to 0, –0.04 or –0.08.

The model is then represented using the following system of ordinary differential equations:

$$\begin{aligned}
\dot{S} &= \mu - (\lambda(t) + \mu)S \\
\dot{E_1} &= \lambda(t)S - (2\sigma + \mu)E_1 \\
\dot{E_2} &= 2\sigma E_1 - (2\sigma + \mu)E_2 \\
\dot{I_1} &= 2\sigma E_2 - (2\gamma + \mu)I_1 \\
\dot{I_2} &= 2\gamma I_1 - (2\gamma + \mu)I_2 \\
\dot{R} &= 2\gamma I_2 - \mu R
\end{aligned}$$

The seasonal force of infection is given by:

$$\lambda(t) = R_0 \gamma S_1(t) S_2(t)(I_1 + I_2)$$

Briefly, individuals started at the susceptible to varicella compartment (S). Next, susceptible individuals entered the exposed compartment E with a seasonal force of infection λ (per susceptible rate of infection, reproduction number, 10) [16]. Then, exposed individuals become infectious at a rate σ ($1/\sigma$ = average latent period, 14 days) [17], enter the infectious compartment I, and then move to the R recovered compartment at a rate γ ($1/\gamma$ = average recovery period, 7 days) [17].

For all simulations, the seasonal model was initialized at the values of the endemic equilibrium of the corresponding seasonally unforced model. The model was then simulated for 200 years, and the last year of the simulation was used for assessing the seasonality of weekly cases.

S2.1. Numerical implementation

S2.2. Numerical implementation R version 4.1.2 (2021-11-01) was used for all analyses. The climate data were obtained using the packages “ncdf4”, “chirsp”, “humidity”, and “kgc” [18–21]. Colombia map figures were created using the package “colmaps” [22]. The package “mgcv” was used for all the GAMs estimations [23]. The dissimilarity matrices, clusters, and mantel tests were obtained with the packages “TSclust” and “vegan” [24,25]. The simulations were performed using the “pomp” package [26].

S3 Supplementary figures

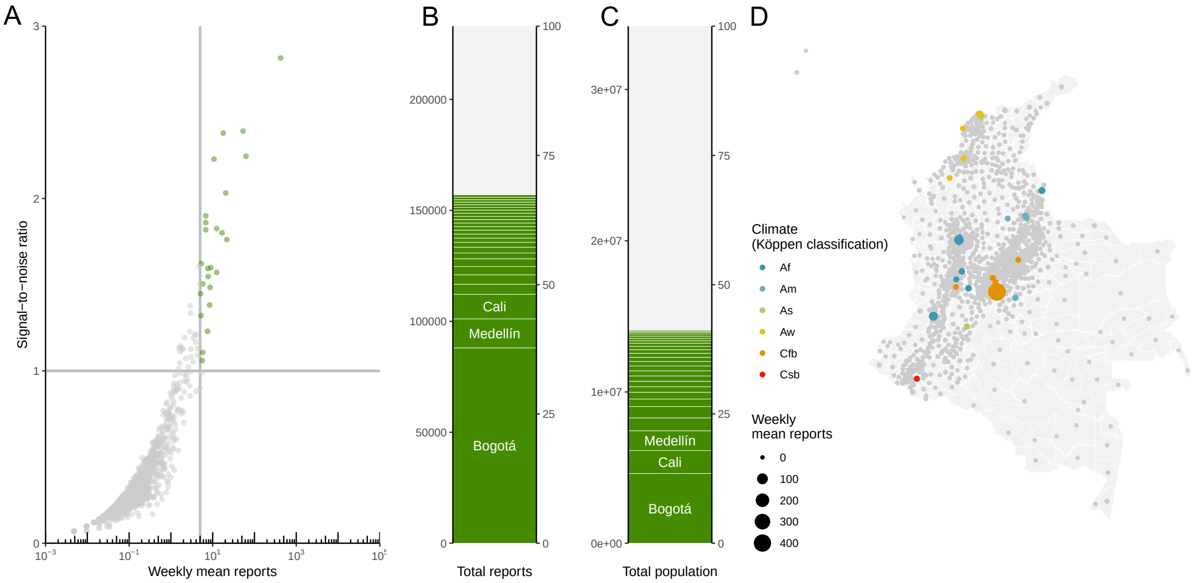

Supplementary figure 1. Selection of municipalities according to varicella reports and signal-to-noise ratio. For definiteness, we selected the municipalities with a signal-to-noise ratio (mean to standard deviation ratio of the weekly reports) over one and an average of at least five cases/week. (A) 25 municipalities met the criterion and collectively included (B) 67.4% of the total reports of varicella, (C) 41.2% of the total study population (children ≤10 years of age). (D) Map of included municipalities, the 25 municipalities included 6 of the 17 Köppen–Geiger climatic classifications most commonly found in the tropics.

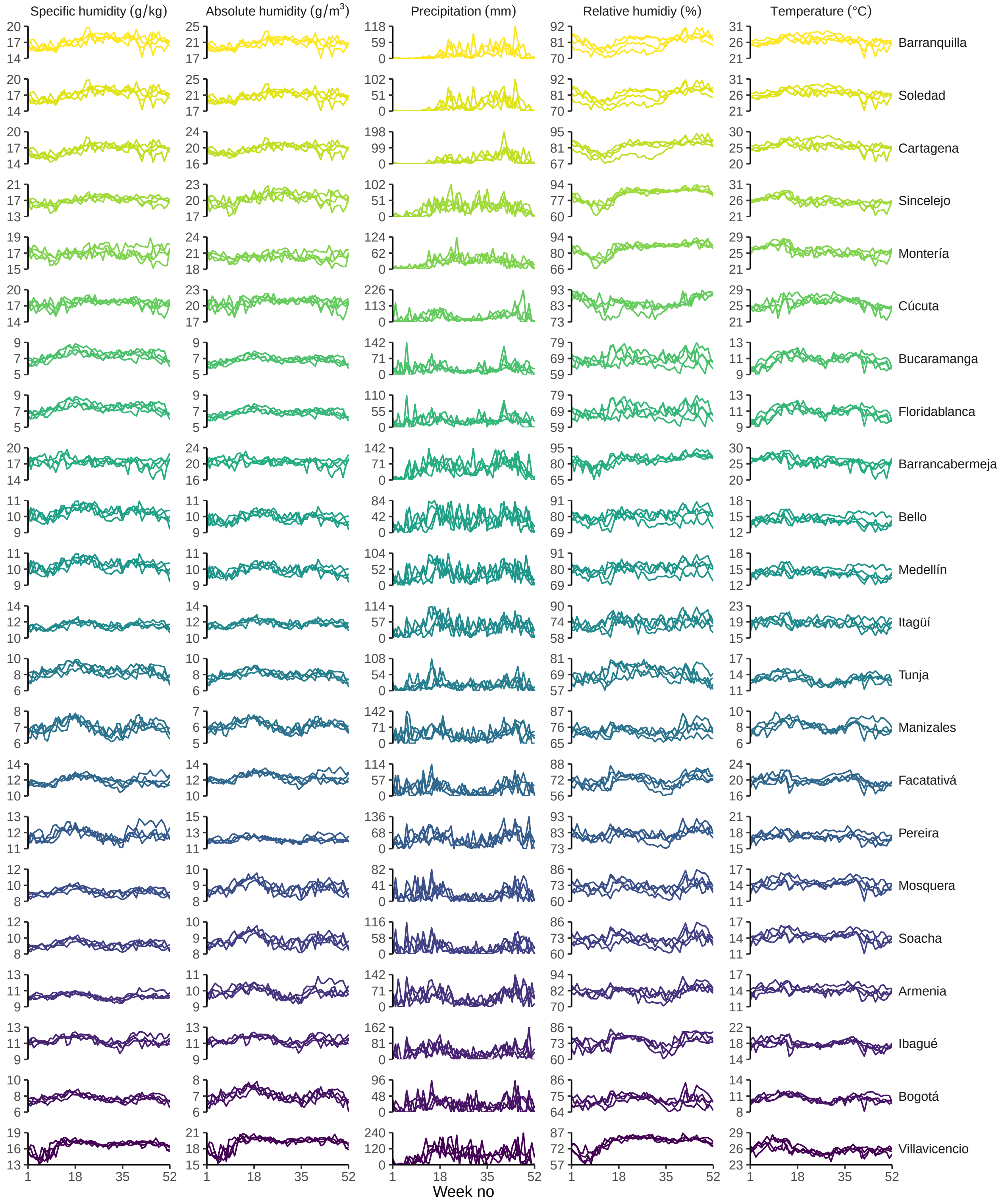

Supplementary figure 2. The seasonal climate in municipalities of Colombia. For the study period (2011–2014), we extracted the weekly specific humidity (expressed in g/kg), absolute humidity (g/m^3), relative humidity (%), and temperature (°C) from the North America Regional Analysis (NARR) [27] and the weekly precipitation (mm) from the Climate Hazards Group InfraRed Precipitation with Station database (CHIRPS) [28].

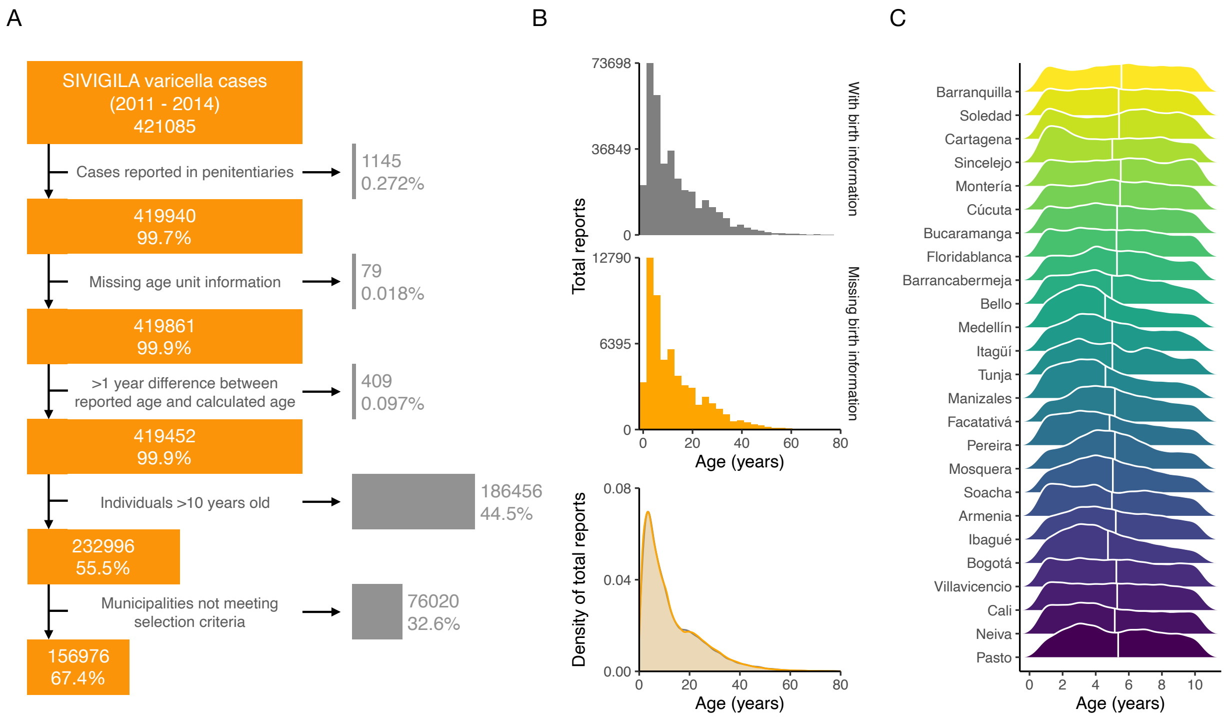

Supplementary figure 3. Data flow chart on varicella reports. We accessed varicella cases for the study period (2011–2014) from the *Sistema Nacional de Vigilancia en Salud Pública* (SIVIGILA). (A) Data flow chart. (B) For 60,257 cases that reported age but no birthdate information, we assumed that the age was not misclassified as their distribution was not different from the cases with birthdate data. (C) The age distribution of varicella infections across municipalities in Colombia in children ≤10 years of age. The vertical white line represents the mean age at infection.

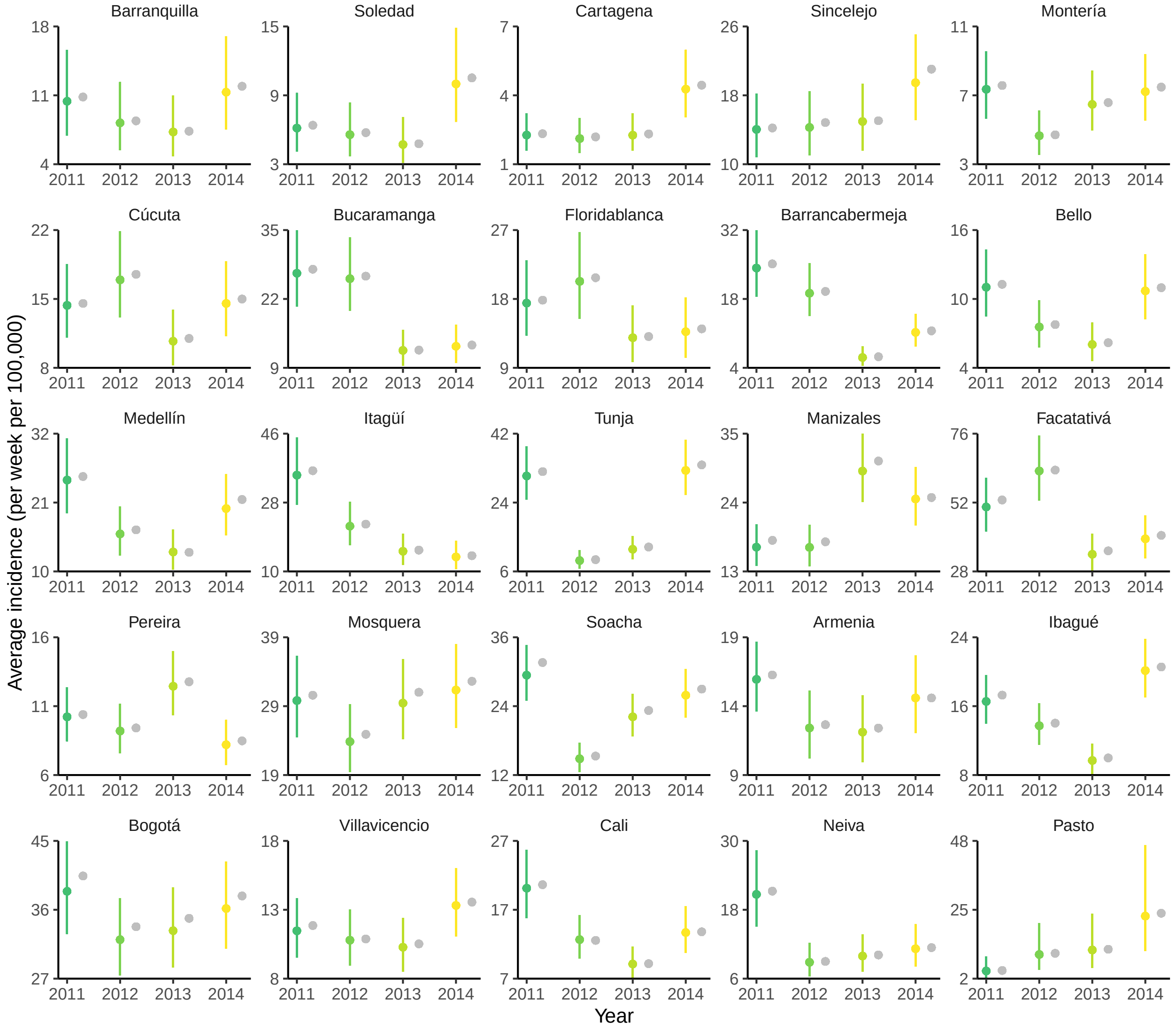

Supplementary figure 4. Predicted average incidence (per week per 100,000) in each municipality of Colombia. The colored points (and colored lines) represent the point estimates (and 95% confidence intervals) of the average weekly incidence for every year and every municipality, estimated from the GAMs. The gray points indicate the observed average weekly incidence estimated directly from the data.

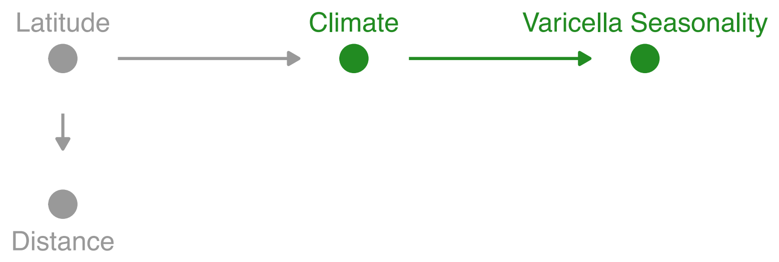

Supplementary figure 5. Assumed directed acyclic graph (DAG) on the spatial effect of climate on the varicella seasonality. In this DAG, the causal effect of latitude is mediated by climate; the effect of distance between municipalities is non-causal, but an association results from the confounding effect of latitude and climate. Hence, this DAG results in the testable implications that, after conditioning on climate, varicella seasonality is no longer associated with either latitude or distance.

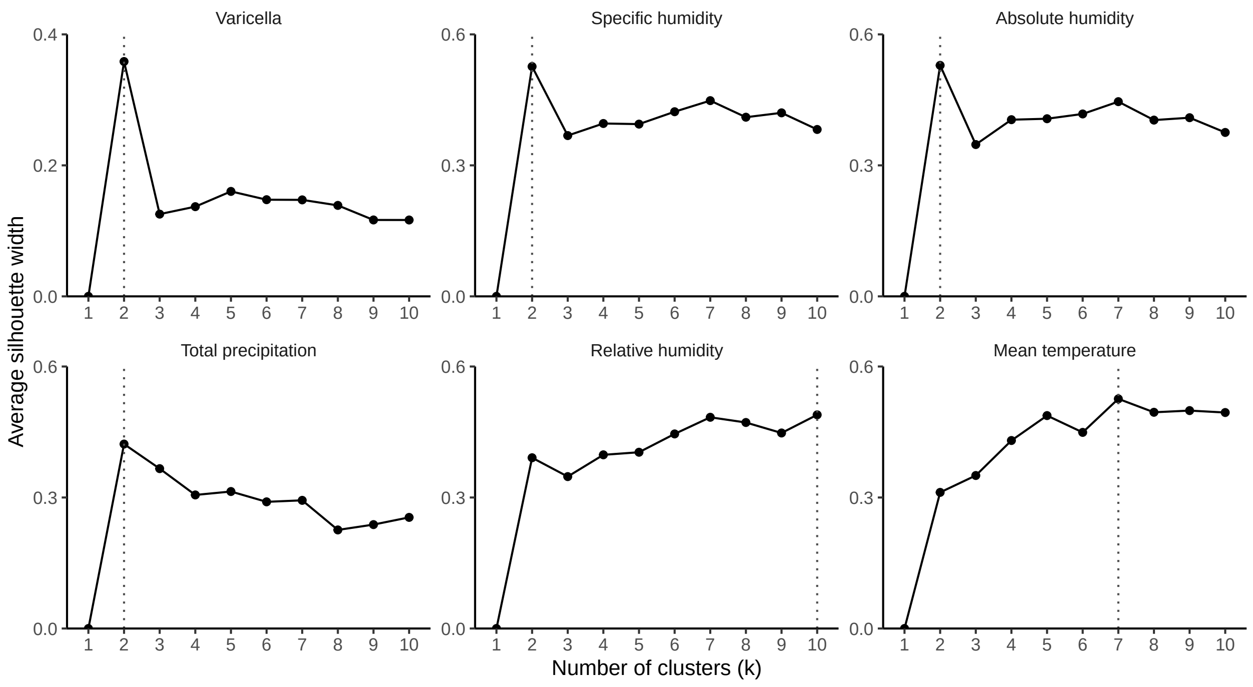

Supplementary figure 6. The optimal number of clusters for varicella and each climatic variable. Average silhouette widths were calculated for 1 to 10 clusters per variable. The dotted line shows the selected number of clusters.

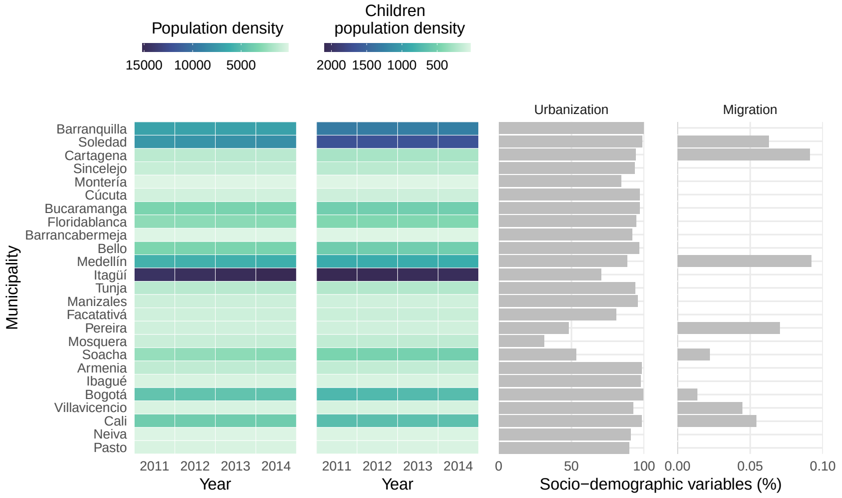

Supplementary figure 7. Socio-demographic characteristics of Colombian municipalities. Population density (people per km^2) and population density of children ≤10 years of age (per km^2) for each municipality of Colombia from 2011–2014. Percentage of total varicella cases reported among children in urban areas and children with migrant status during the same period.

Supplementary table 1. Correlation between the spatial heterogeneity of varicella seasonality and that of socio-demographic factors. The correlations were calculated using Mantel tests on the dissimilarity matrices calculated using Euclidean distances between the time series of paired municipalities.

Variable tested	Mantel statistic	p-value
Population density	-0.114	0.814
Children ≤10 density	-0.133	0.827
Urbanization	-0.096	0.705
Migration	-0.085	0.689

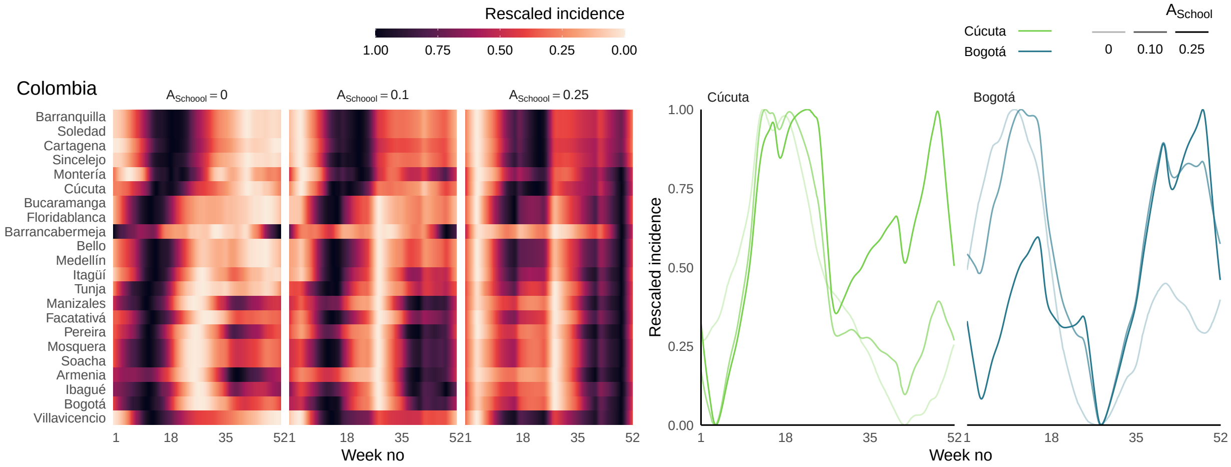

Supplementary figure 8. Impact of term-time forcing on varicella seasonality across Colombia. Predicted varicella incidence (rescaled so that 0 is the minimum and 1 is the maximum number of cases per municipality) for all municipalities in Colombia using a constant humidity effect (–0.08). From bottom to top, the municipalities are ordered by increasing latitude.

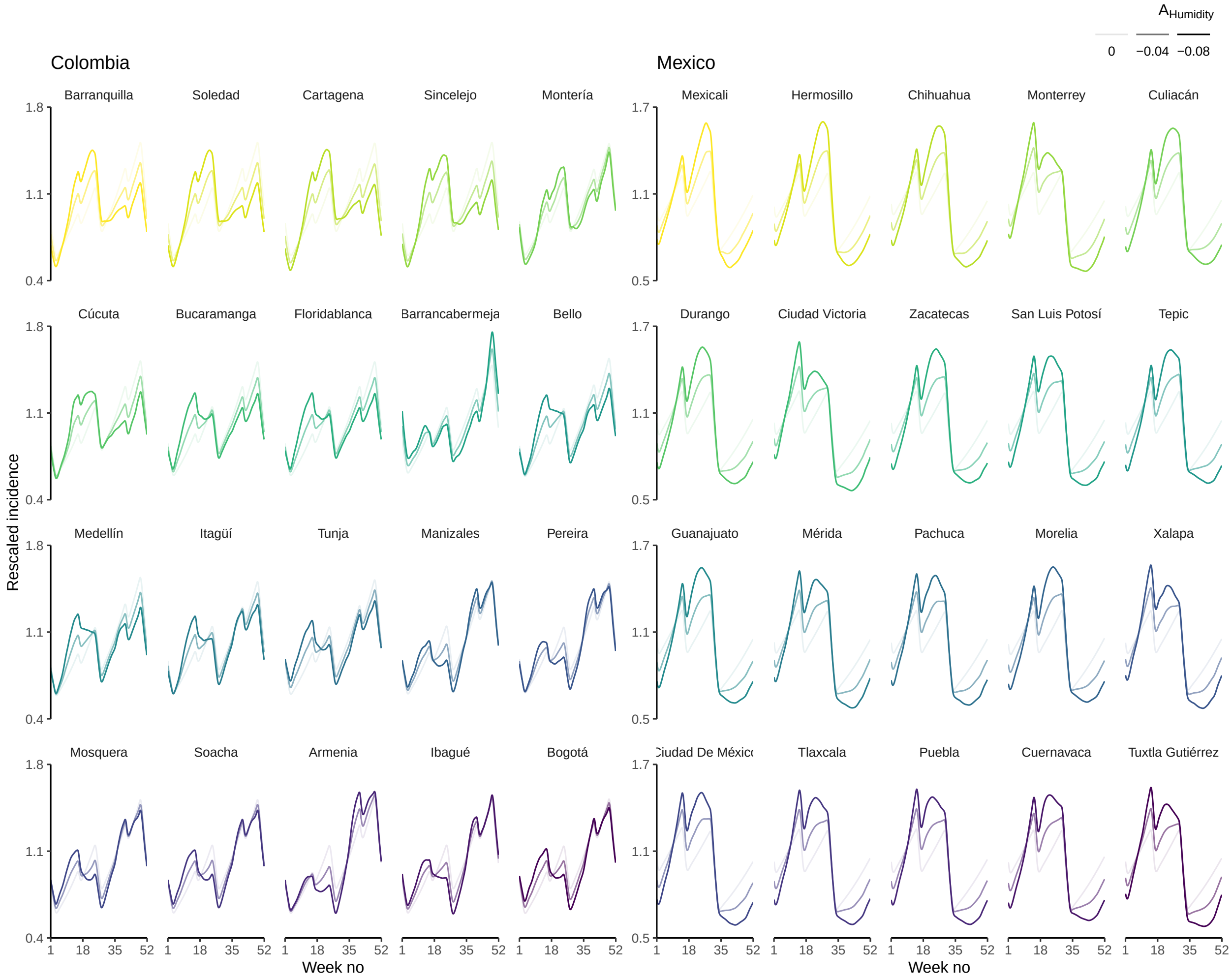

Supplementary figure 9. Predicted seasonality of varicella across Colombia and Mexico. The lines represent the weekly incidence of varicella (rescaled to have a yearly average of 1) predicted from a transmission model incorporating term-time forcing and different levels of specific humidity forcing.

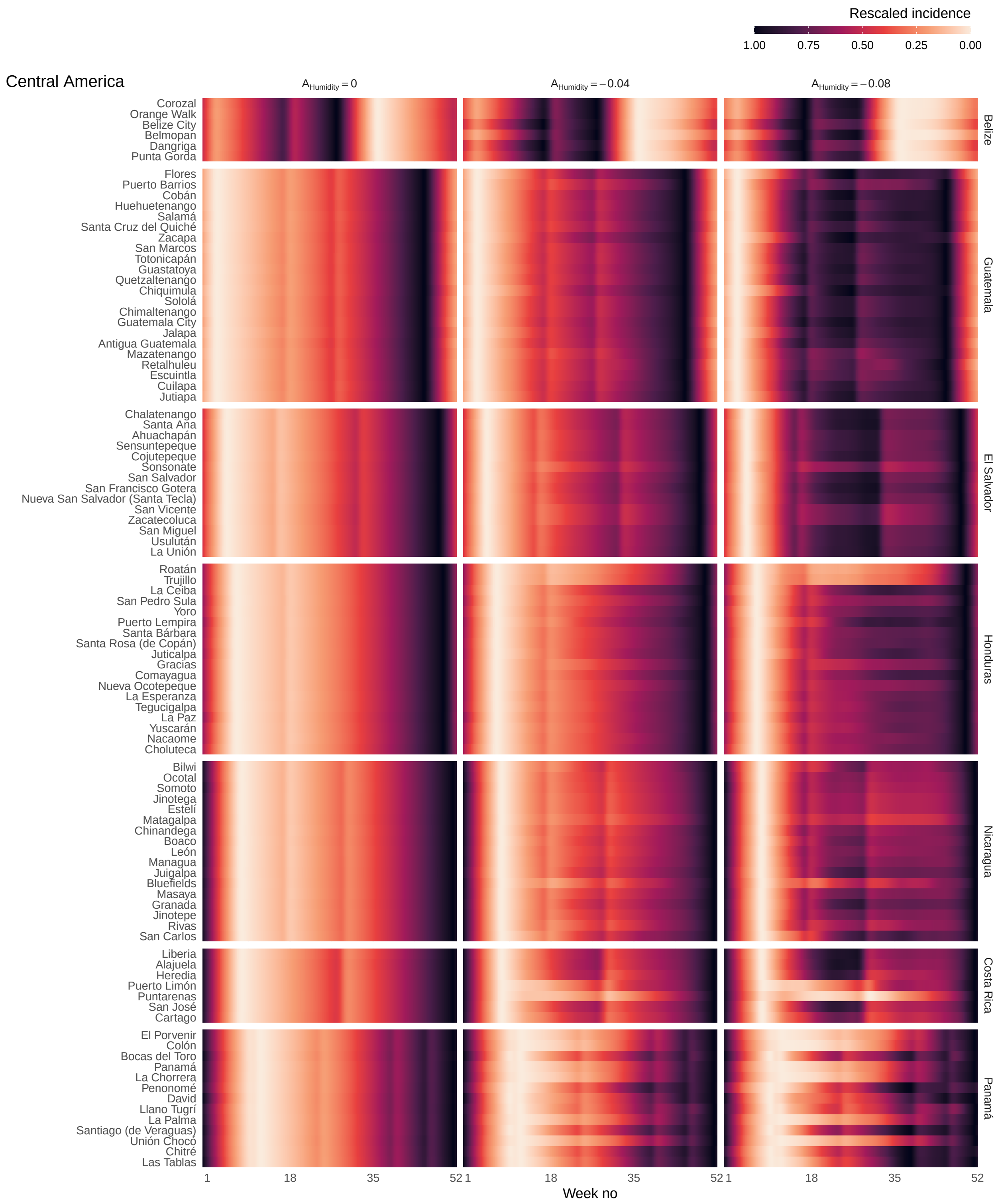

Supplementary figure 10. Predicted seasonality of varicella in Central American countries. The heatmap represents the incidence of varicella (rescaled to range between 0 and 1) predicted from a transmission model incorporating term-time forcing and specific humidity forcing (with increasing amplitude of forcing from left to right). In every country, the municipalities are ordered by increasing latitude from bottom to top.

References

1. Por medio de la cual se crea la Ley de Transparencia y del Derecho de Acceso a la Información Pública Nacional y se dictan otras disposiciones [Internet]. Ley 1712 de 2014. Available from: https://www.funcionpublica.gov.co/eva/gestornormativo/norma.php?i=56882

2. Por la cual se dictan disposiciones generales para la protección de datos personales [Internet]. Ley 1581 de 2012. Available from: https://www.funcionpublica.gov.co/eva/gestornormativo/norma.php?i=49981

3. Instituto Nacional de Salud. Microdatos Sistema Nacional de Vigilancia en Salud Pública de Colombia [Internet]. Portal SIVIGILA. [cited 2022 May 16]. Available from: http://portalsivigila.ins.gov.co/Paginas/Buscador.aspx

4. Aguilar W, Benavides M, Lozano Falla NR. Manual del Usuario Software SIVIGILA [Internet]. Instituto Nacional de Salud, Ministerio de Salud, Colombia; **2010**. Available from: https://www.minsalud.gov.co/salud/Paginas/SIVIGILA.aspx

5. Departamento Administrativo Nacional de Estadística. Estadísticas por tema: Demografía y población [Internet]. Portal DANE. [cited 2022 Jul 23]. Available from: https://www.dane.gov.co/index.php/estadisticas-por-tema/demografia-y-poblacion

6. Instituto de Hidrología, Meteorología y Estudios Ambientales. Clima de Colombia [Internet]. Portal IDEAM. [cited 2022 Oct 19]. Available from: http://www.ideam.gov.co/web/tiempo-y-clima/clima

7. Ministerio de Educación Nacional. Micrositios Preescolar, Básica y Media de Colombia [Internet]. Portal MEN. [cited 2022 Dec 5]. Available from: https://www.mineducacion.gov.co/portal/micrositios-preescolar-basica-y-media/

8. Ministerio de Educación. Ministerio de Educación de Panamá [Internet]. MEDUCA. [cited 2022 Dec 5]. Available from: https://www.meduca.gob.pa/

9. Ministerio de Educación Pública. Ministerio de Educación Pública de Costa Rica [Internet]. MEP. [cited 2022 Dec 5]. Available from: https://www.mep.go.cr/

10. Ministerio de Educación. Ministerio de Educación de Nicaragua [Internet]. MINED. [cited 2022 Dec 5]. Available from: https://www.mined.gob.ni/

11. Secretaría de Educación. Secretaría de Educación de Honduras [Internet]. SE. [cited 2022 Dec 5]. Available from: https://www.se.gob.hn/

12. Ministerio de Educación. Ministerio de Educación de El Salvador [Internet]. MINED. [cited 2022 Dec 5]. Available from: https://www.mined.gob.sv/

13. Ministerio de Educación. Ministerio de Educación de Guatemala [Internet]. MINEDUC. [cited 2022 Dec 5]. Available from: https://www.mineduc.gob.gt/portal/index.asp

14. Ministry of Education, Culture, Science and Technology. Ministry of Education, Culture, Science and Technology of Belize [Internet]. MOECST. [cited 2022 Dec 5]. Available from: https://www.moecst.gov.bz/

15. Eames KTD, Tilston NL, Brooks-Pollock E, Edmunds WJ. Measured dynamic social contact patterns explain the spread of H1N1v influenza. PLoS Comput Biol. **2012**; 8(3):e1002425.

16. Leung NHL. Transmissibility and transmission of respiratory viruses. Nat Rev Microbiol. **2021**; 19(8):528–545.

17. Brisson M, Edmunds WJ, Gay NJ, Law B, De Serres G. Modelling the impact of immunization on the epidemiology of varicella zoster virus. Epidemiol Infect. **2000**; 125(3):651–669.

18. Cai J. humidity: Calculate Water Vapor Measures from Temperature and Dew Point [Internet]. **2019**. Available from: https://github.com/caijun/humidity

19. Pierce D. ncdf4: Interface to Unidata netCDF (Version 4 or Earlier) Format Data Files [Internet]. **2021**. Available from: https://CRAN.R-project.org/package=ncdf4

20. Bryant C, Wheeler NR, Rubel F, French RH. kgc: Koeppen-Geiger Climatic Zones [Internet]. **2017**. Available from: https://CRAN.R-project.org/package=kgc

21. Sousa K de, Sparks AH, Ashmall W, Etten J van, Solberg SØ. chirps: API Client for the CHIRPS Precipitation Data in R. The Journal of Open Source Software. The Open Journal; **2020**; 5(51):2419.

22. Moreno D. colmaps: Colombian maps. **2015**.

23. Wood SN. Generalized Additive Models: An Introduction with R. 2nd ed. Chapman and Hall/CRC; **2017**.

24. Montero P, Vilar JA. TSclust: An R Package for Time Series Clustering. J Stat Softw. **2014**; 62(1):1–43.

25. Oksanen J, Blanchet FG, Friendly M, et al. vegan: Community Ecology Package [Internet]. **2020**. Available from: https://CRAN.R-project.org/package=vegan

26. King AA, Nguyen D, Ionides EL. Statistical Inference for Partially Observed Markov Processes via the R Package pomp [Internet]. J. Stat. Softw. **2016** [cited 2022 Aug 11]. p. 1–43. Available from: http://dx.doi.org/10.18637/jss.v069.i12

27. Mesinger F, DiMego G, Kalnay E, et al. North American Regional Reanalysis. Bull Am Meteorol Soc. American Meteorological Society; **2006**; 87(3):343–360.

28. Funk C, Peterson P, Landsfeld M, et al. The climate hazards infrared precipitation with stations–a new environmental record for monitoring extremes. Sci Data. **2015**; 2:150066.
